# Supplementary material for: Numerosity tuning in human association cortices and local image contrast representations in early visual cortex
Source: Nat Commun. 2022 Mar 15;13:1340. doi: 10.1038/s41467-022-29030-z (PMC8924234; doi:10.1038/s41467-022-29030-z)
Supplement: Supplementary file 2 — Reporting summary [file 41467_2022_29030_MOESM2_ESM.pdf]

nature research

Corresponding author(s): Jacob M. Paul

Last updated by author(s): Feb 2, 2022

Reporting Summary

Nature Research wishes to improve the reproducibility of the work that we publish. This form provides structure for consistency and transparency in reporting. For further information on Nature Research policies, see our Editorial Policies and the Editorial Policy Checklist.

Statistics

For all statistical analyses, confirm that the following items are present in the figure legend, table legend, main text, or Methods section.

n/a	Confirmed	
☐	☒	The exact sample size (*n*) for each experimental group/condition, given as a discrete number and unit of measurement
☐	☒	A statement on whether measurements were taken from distinct samples or whether the same sample was measured repeatedly
☐	☒	The statistical test(s) used AND whether they are one- or two-sided<br>*Only common tests should be described solely by name; describe more complex techniques in the Methods section.*
☐	☒	A description of all covariates tested
☐	☒	A description of any assumptions or corrections, such as tests of normality and adjustment for multiple comparisons
☐	☒	A full description of the statistical parameters including central tendency (e.g. means) or other basic estimates (e.g. regression coefficient) AND variation (e.g. standard deviation) or associated estimates of uncertainty (e.g. confidence intervals)
☐	☒	For null hypothesis testing, the test statistic (e.g. *F*, *t*, *r*) with confidence intervals, effect sizes, degrees of freedom and *P* value noted<br>*Give P values as exact values whenever suitable.*
☒	☐	For Bayesian analysis, information on the choice of priors and Markov chain Monte Carlo settings
☒	☐	For hierarchical and complex designs, identification of the appropriate level for tests and full reporting of outcomes
☐	☒	Estimates of effect sizes (e.g. Cohen's *d*, Pearson's *r*), indicating how they were calculated

Our web collection on statistics for biologists contains articles on many of the points above.

Software and code

Policy information about availability of computer code

Data collection	7T Philips Achieva scanner, PsychToolbox-3
Data analysis	mrVista 2.0 (https://github.com/vistalab/vistasoft), Matlab version 2018b (The Mathworks), Freesurfer 6.0, ITK-SNAP 1.6.0.1, AFNI 19.1.21 (afni.nimh.nih.gov), graphreader.com

For manuscripts utilizing custom algorithms or software that are central to the research but not yet described in published literature, software must be made available to editors and reviewers. We strongly encourage code deposition in a community repository (e.g. GitHub). See the Nature Research guidelines for submitting code & software for further information.

Data

Policy information about availability of data

All manuscripts must include a data availability statement. This statement should provide the following information, where applicable:

- Accession codes, unique identifiers, or web links for publicly available datasets
- A list of figures that have associated raw data
- A description of any restrictions on data availability

Ethical constraints prevent us from sharing the medical imaging data sets (MRI scans) generated in the current study to public repositories. The structure of the brain is unique to the individual participant, in theory allowing the participant to be identified from these images, which may also contain medically sensitive findings. This is an interpretation of the EU’s General Data Protection Regulation (GDPR) for medical images including MRI data. These raw data sets are available from the corresponding author upon reasonable request, depending on agreements not to share these data publicly. Model parameters underlying all statistical analyses and response data time-series for all model fitting are publicly available at the following DOIs: visual field mapping response model parameters (https://doi.org/10.6084/m9.figshare.17294219); visual field mapping response time-series (https://doi.org/10.6084/m9.figshare.17294060); monotonic/tuned numerosity, aggregate Fourier power, and non-numerical cues response model parameters (https://doi.org/10.6084/m9.figshare.17294390); numerosity response time-series

(https://doi.org/10.6084/m9.figshare.17294342). Data from participants P1-P5 were included in a previous study (Harvey & Dumoulin, 2017, Nature Human Behaviour), although we use updated preprocessing protocols here. Source Data plotted in the Figures are provided with this paper.

Field-specific reporting

Please select the one below that is the best fit for your research. If you are not sure, read the appropriate sections before making your selection.

☐ Life sciences ☒ Behavioural & social sciences ☐ Ecological, evolutionary & environmental sciences

For a reference copy of the document with all sections, see nature.com/documents/nr-reporting-summary-flat.pdf

Behavioural & social sciences study design

All studies must disclose on these points even when the disclosure is negative.

Study description	Quantitative - functional magnetic resonance imaging (fMRI)
Research sample	We acquired fMRI data from eleven participants (aged 25–39 years, one female, one left-handed). All had normal or corrected-to-normal visual acuity, good mathematical abilities and were well educated. The rationale for selecting a research sample of healthy neurotypical participants is that we were interested in studying normal brain function.
Sampling strategy	We acquired fMRI data from a convenience sample of 11 researchers (aged 25–39 years, one female, one left-handed). Six of these were members of our lab and associated labs, five were graduate students from elsewhere in Utrecht University and University Medical Center Utrecht recruited through advertising. No sample size calculation was performed, as all data were originally collected for other studies (Harvey & Dumoulin, 2017, Nature Human Behaviour; Harvey, Dumoulin, Fracasso & Paul, 2020, Current Biology). The data analysis is primarily on an individual participant level, and provides high statistical significance in each individual participant. Multiple participants used to demonstrate that results are reproducible across participants. In the studies for which the data were originally collected, sample sizes were chosen based on the sample sizes that had previously been used to convincingly demonstrate reproducibility in experiments with similar designs throughout the field.
Data collection	We acquired MRI data on a 7T Philips Achieva scanner. No one else was present besides the participant(s) and the researchers during data collection, and the researchers were not blinded because there were no experimental conditions.
Timing	Data for participants 1 - 5 were collected from 03/04/2013 to 23/04/2014. Data for participants 6 - 11 were collected from 21/11/2017 to 18/07/2019.
Data exclusions	Data from all scanned participants was included. However, we acquired data from the whole brain, although only a small set of regions was analyzed, as in all fMRI experiments. Most locations in the brain do not respond to the changes in numerosity. We first excluded from analysis any recording sites (voxels) that lay outside the gray matter, which was pre-established and a standard procedure in the field. After the fit of population receptive field models and monotonic response models, we excluded from further analyses voxels where the numerosity preference was beyond the range of the presented stimuli or when the variance explained of either model was lower than 20% in cross-validated data. This was in total several million excluded voxels.
Non-participation	No participants dropped out/declined participation.
Randomization	Participants were not allocated into experimental groups.

Reporting for specific materials, systems and methods

We require information from authors about some types of materials, experimental systems and methods used in many studies. Here, indicate whether each material, system or method listed is relevant to your study. If you are not sure if a list item applies to your research, read the appropriate section before selecting a response.

Materials & experimental systems

n/a	Involved in the study	
☒	☐	Antibodies
☒	☐	Eukaryotic cell lines
☒	☐	Palaeontology and archaeology
☒	☐	Animals and other organisms
☐	☒	Human research participants
☒	☐	Clinical data
☒	☐	Dual use research of concern

Methods

n/a	Involved in the study	
☒	☐	ChIP-seq
☒	☐	Flow cytometry
☐	☒	MRI-based neuroimaging

Human research participants

Policy information about studies involving human research participants

Population characteristics	See above
Recruitment	Participants were recruited from Utrecht University and Utrecht Medical Center either in response to recruitment posters for the study or from the lab. Although there are potential self-selection biases in using a convenient sample, such a recruitment strategy in this case is likely to self-select for an interest in neuroimaging or brain function more generally, which is unlikely to have any impact on an individuals' brain activity and therefore no impact on our results.
Ethics oversight	All experimental procedures were approved by the ethics committee of University Medical Center Utrecht.

Note that full information on the approval of the study protocol must also be provided in the manuscript.

Magnetic resonance imaging

Experimental design

Design type	Task fMRI
Design specifications	The numerosities one through seven were first presented in ascending order, with numerosity changing every 4200 ms (two TRs). Within this period, a numerosity pattern was shown for 300 ms, alternating with 400 ms of gray background, repeated six times. Each numerosity pattern had items drawn in new, random positions. These short presentations prevented participants from counting. Following this, twenty items were presented in the same way for eight TRs (16.8s, 24 presentations of a pattern). These periods of twenty items served to distinguish between very small and very large tuning widths. Then numerosities one through seven were presented as before, but in descending order, followed by another long period of twenty items. This cycle was repeated 4 times in each scanning run. Each functional run acquired 182 images (382.2s) of which the first six (12.6s) were discarded to ensure a steady signal state. In each scan session, we acquired six to eight repeated runs in one stimulus configuration, plus a top-up scan with the opposite phase-encoding direction to correct for image distortion in the gradient encoding direction, and a T1-weighted anatomical image with the same resolution, position and orientation as the functional data. Different stimulus configurations were tested in different sessions. For visual field mapping, a bar filled with a moving checkerboard pattern stepped across a 6.35° (radius) circle in the display center in eight (cardinal and diagonal) directions.
Behavioral performance measures	During visual field mapping the central fixation cross changed color (red/green) and on 10% of trials during presentation of visual numerosity stimuli the dots changed color (black/white). Participants were instructed to press a button when a color change occurred to confirm they were paying attention to the stimuli and remained awake throughout scanning.

Acquisition

Imaging type(s)	Functional
Field strength	7 Tesla
Sequence & imaging parameters	We acquired T1-weighted anatomical scans and T2*-weighted functional images using a 32-channel head coil at a resolution of 1.77×1.77×1.75 mm, with 41 interleaved slices of 128×128 voxels. The resulting field of view was 227×227×72 mm. TR was 2100 ms, TE was 25 ms, and flip angle was 70 degrees. We used a single shot gradient echo sequence with SENSE acceleration factor 3.0 and anterior-posterior encoding.
Area of acquisition	Whole brain scan (excluding anterior frontal and temporal lobes, where 7T fMRI has low response amplitudes and large spatial distortions).

Diffusion MRI ☐ Used ☒ Not used

Preprocessing

Preprocessing software	Freesurfer 6.0, ITK-SNAP 1.6.0.1, AFNI 19.1.21 (afni.nimh.nih.gov)
Normalization	Analyses were performed in each participants' native space. Data were not normalized because there we no group comparisons or experimental conditions, and we were interested in fine-scale structures of individual healthy brains which would have been obscured with group normalization to a single template.
Normalization template	Analyses were performed in each participants' native space. Data were not normalized because there we no group comparisons or experimental conditions, and we were interested in fine-scale structures of individual healthy brains which would have been obscured with group normalization to a single template.
Noise and artifact removal	Functional scans were corrected for head movement and motion with two series of images that were acquired using opposing phase-encoding directions, with transformations calculated using AFNI (3dvolreg, 3dQwarp, 3dNwarpApply). No other spatial or temporal smoothing procedures were applied.
Volume censoring	Not used

Statistical modeling & inference

Model type and settings	Population receptive field modeling and general linear models.
Effect(s) tested	Model fits with variance explained higher than 20%.

Specify type of analysis: [x] Whole brain [ ] ROI-based [ ] Both

Statistic type for inference (See Eklund et al. 2016)	Voxel-wise
Correction	The voxel-wise population receptive field model and monotonic response model fits were not corrected for multiple comparisons, but all following statistics on visual field maps were.

Models & analysis

n/a	Involved in the study
[x]	[ ] Functional and/or effective connectivity
[x]	[ ] Graph analysis
[ ]	[x] Multivariate modeling or predictive analysis

Multivariate modeling and predictive analysis	Population receptive field modeling and general linear models
